# Supplementary material for: The core metabolome and root exudation dynamics of three phylogenetically distinct plant species
Source: Nat Commun. 2023 Mar 24;14:1649. doi: 10.1038/s41467-023-37164-x (PMC10039077; doi:10.1038/s41467-023-37164-x)
Supplement: Supplementary file 1 — Supplementary Information [file 41467_2023_37164_MOESM1_ESM.pdf]

Supplementary Information for

The core metabolome and root exudation dynamics of three phylogenetically distinct plant species

Sarah McLaughlin[1,2,3], Kateryna Zhalnina[1,2], Suzanne Kosina[1], Trent R. Northen[1,2] *, Joelle Sasse[1,2, 3]*

Correspondence to: trnorthen@lbl.gov, jsasse@botinst.uzh.ch

Supplementary Figures

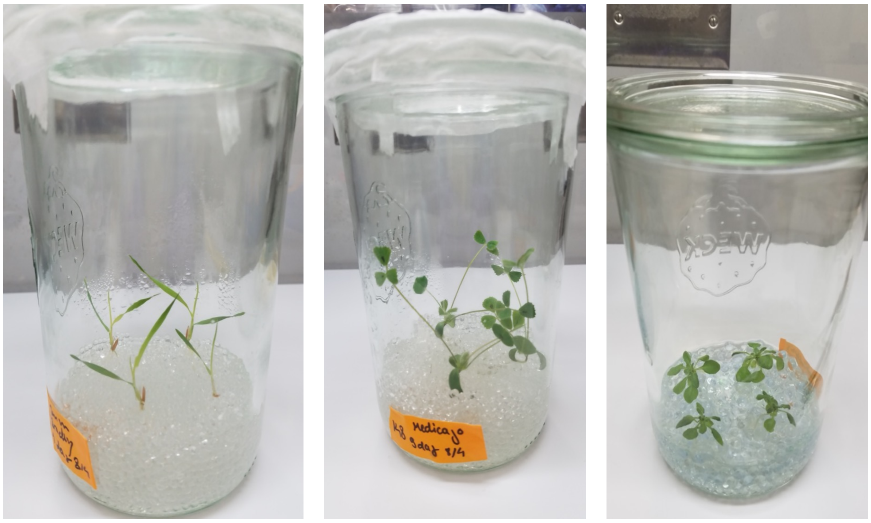

B. distachyon 14 dag | *M. truncatula* 21 dag | *A. thaliana* 21 dag

Supplementary Figure 1: Experimental setup
B. distachyon, *M. truncatula*, and *A. thaliana* were grown in a hydroponic setup. Jars were filled with glass beads and 0.5 MS medium. Plants were germinated on plates, and transferred to jars when germinated (see methods section for detailed description). For each condition, several jars were set up for exudate sampling (data points presented), and empty jars were prepared as negative controls. dag: days after germination.

a *B. distachyon*

	0.5 h	2 h	4 h	1 d
**2 h**	0			
**4 h**	5	0		
**1 d**	14	14	0	
**4 d**	30	30	25	21

- 63 compounds
- 23 compounds (37%) significantly different

b *A. thaliana*

	0.5 h	2 h	4 h	1 d
**2 h**	0			
**4 h**	2	2		
**1 d**	10	10	5	
**4 d**	52	49	43	32

- 63 compounds
- 33 compounds (52%) significantly different

c *M. truncatula*

	0.5 h	2 h	4 h	1 d
**2 h**	2			
**4 h**	2	0		
**1 d**	0	2	0	
**4 d**	13	13	8	6

- 63 compounds
- 10 compounds (16%) significantly different

Supplementary Figure 2: Significant changes in metabolite abundance over time
Significantly different metabolites in percent in pairwise comparisons in the different timepoints of exudate collection for *B distachyon* (**A**), *A. thaliana* (**B**), and *M. truncatula* (**C**) (Anova/Tukey test, $p < 0.05$). Principal component plots of the data are displayed in Figure 1, and changes of single metabolites in Supplementary Figure 3. Colored by high (dark green) to low (light green) number of metabolites different. Number of jars with 3-5 plants: 3-4 for each timepoint. One representative experiment out of three total is displayed.

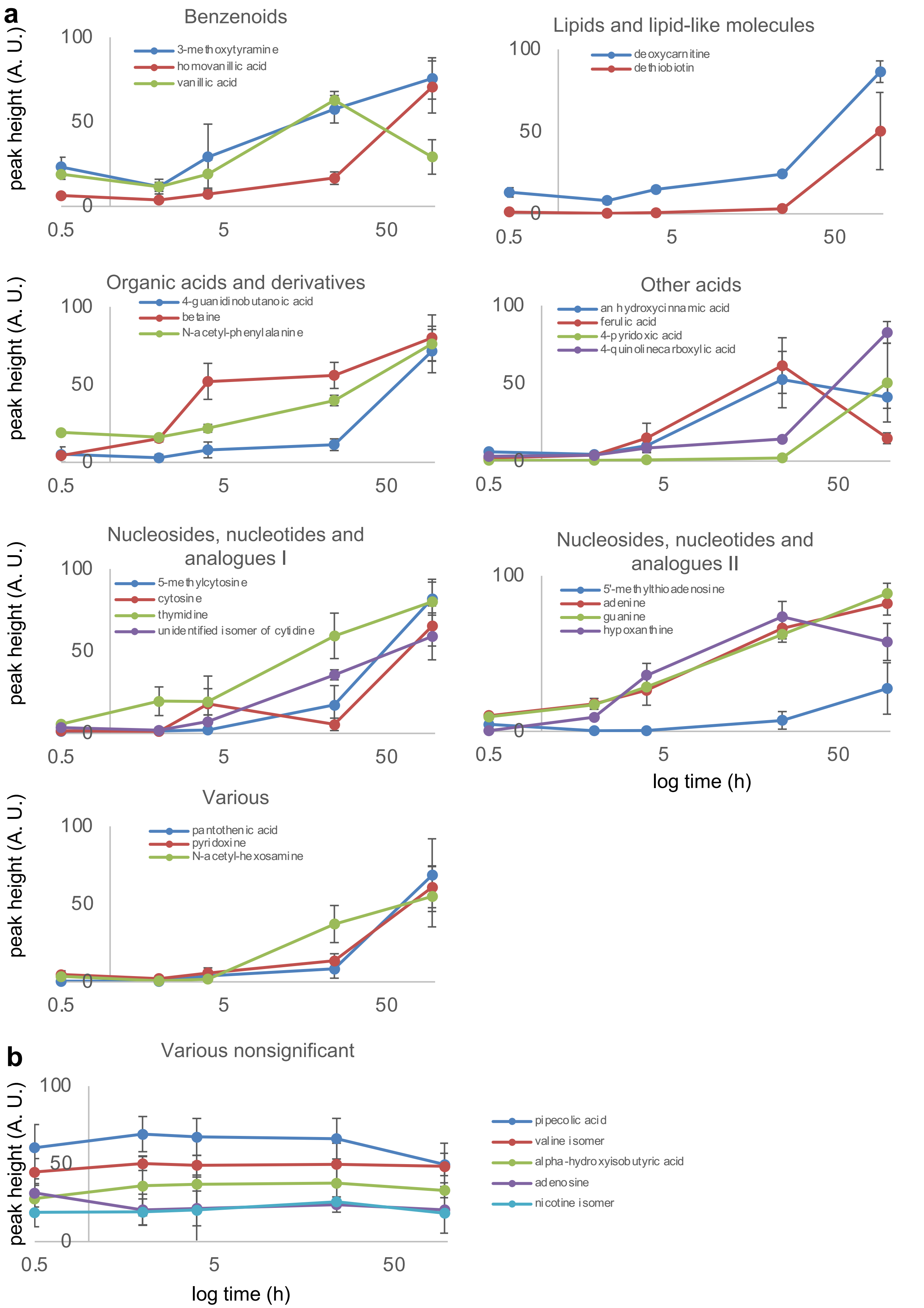

Supplementary Figure 3: *B. distachyon* root exudation dynamics of single metabolites

Selected metabolite abundance changes over time. Displayed are scaled peak heights in arbitrary units (A.U.), and time on a logarithmic scale. Timepoints: 0.5 h, 2 h, 4 h, 1 d, 4 d. **A**, selected metabolites with statistically different abundances between timepoint 0.5 h and 4 d (Anova/Tukey test, $p < 0.05$), grouped by metabolite class. **B**, selected metabolites without significant abundance changes over time. Data are averages ± S.E.M, n = 3-4 jars with 3-5 plants each. One representative experiment out of three total is displayed.

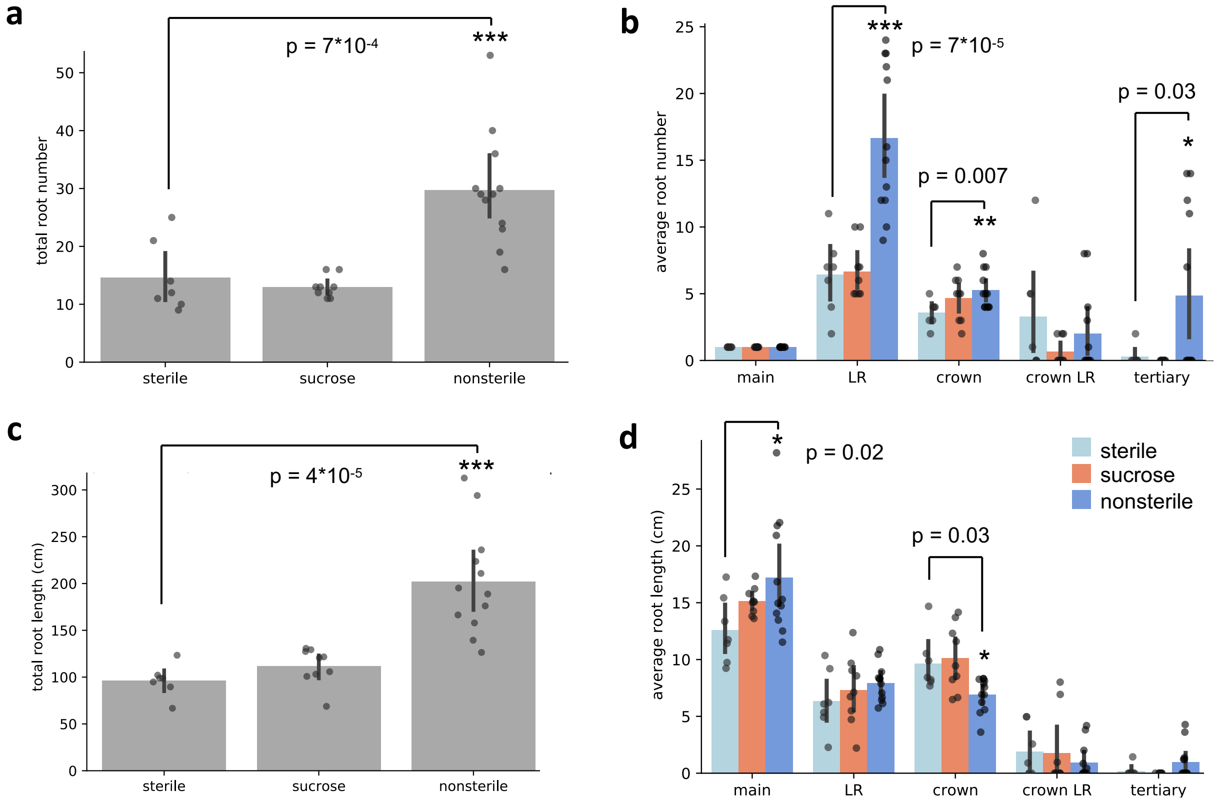

Supplementary Figure 4: Root morphology of *B. distachyon* grown in different conditions

B. distachyon was grown for 3 weeks in sterile, nonsterile, and sucrose-supplemented conditions. Total root number (A) and length (C), and average root number (B) and length (D) per root type and condition are displayed. Data are averages ± S.E.M., n = 15 biologically independent plants, two-sided t-test: * = $p < 0.05$, *** = $p < 0.0005$ (n = 7-12 plants). Tissue weight and exudate profiles of plants is displayed in Figure 2. LR: lateral root.

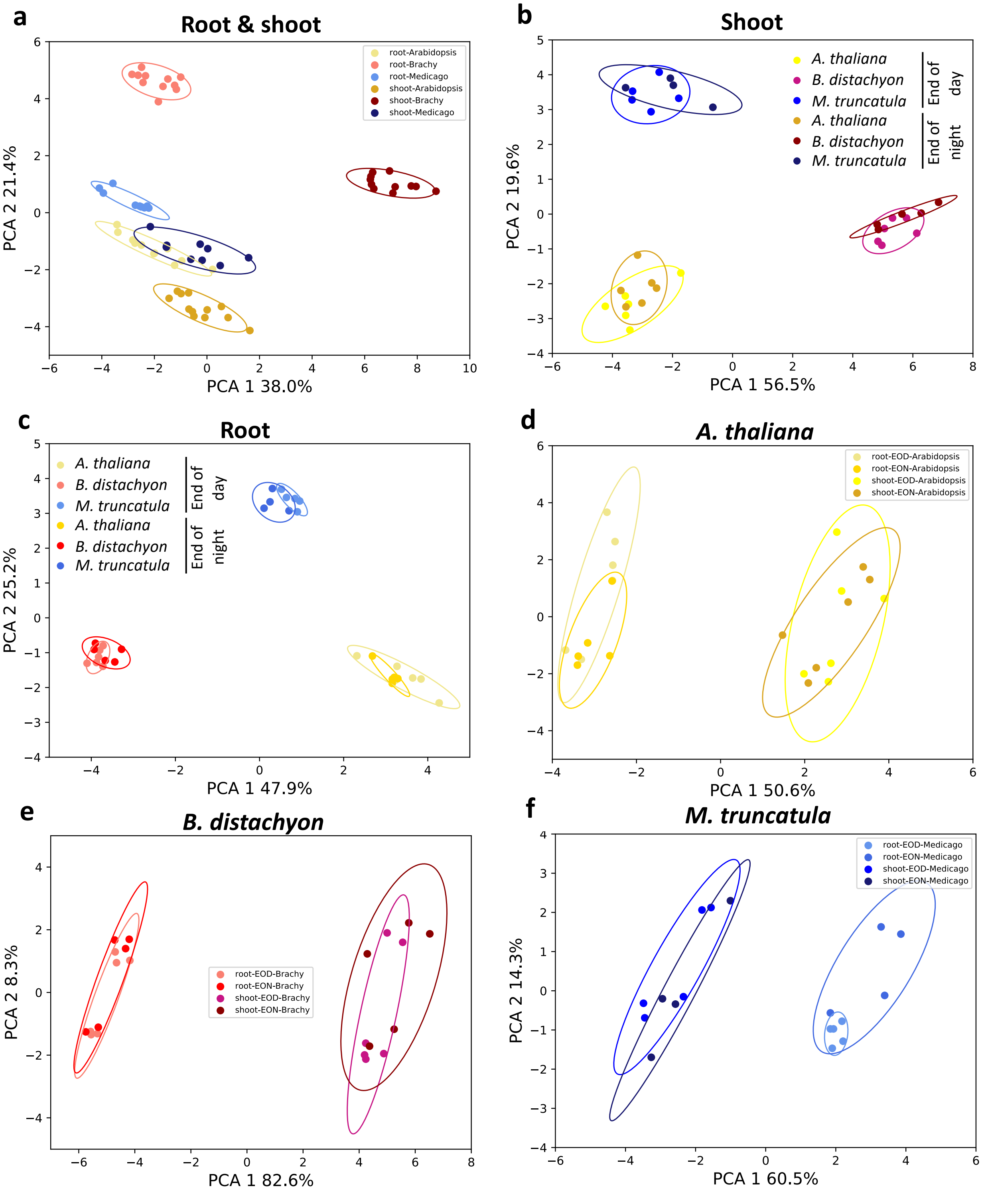

g

	B. distachyon	*A. thaliana*
A. thaliana	67	
M. truncatula	67	39

h

	B. distachyon	*A. thaliana*
A. thaliana	27	
M. truncatula	20	20

i

	Shoot-Root
A. thaliana	33
B. distachyon	19
M. truncatula	76

Supplementary Figure 5: Species-specific metabolites in tissues

Principal component analyses of end of day and end of night root and shoot metabolic profiles of the three species showing clustering of root and shoot metabolite,s respectively (**A**), of shoots (**B**) and roots (**C**) showing differential clustering of the three species, and of the tissues within each species A. thaliana (**D**), *B. distachyon* (**E**), *M. truncatula* (**F**). Percentage of metabolites significantly different (Anova / Tukey test, $p < 0.05$) between shoots (**G**) and roots (**H**) of the three species, as well as between roots and shoots within one species (**I**). Data used is the same as displayed in Figure 5. Total number of metabolites: 143. Number of jars with 3-5 plants: 3-8 for each timepoint. One representative experiment out of three total is displayed.

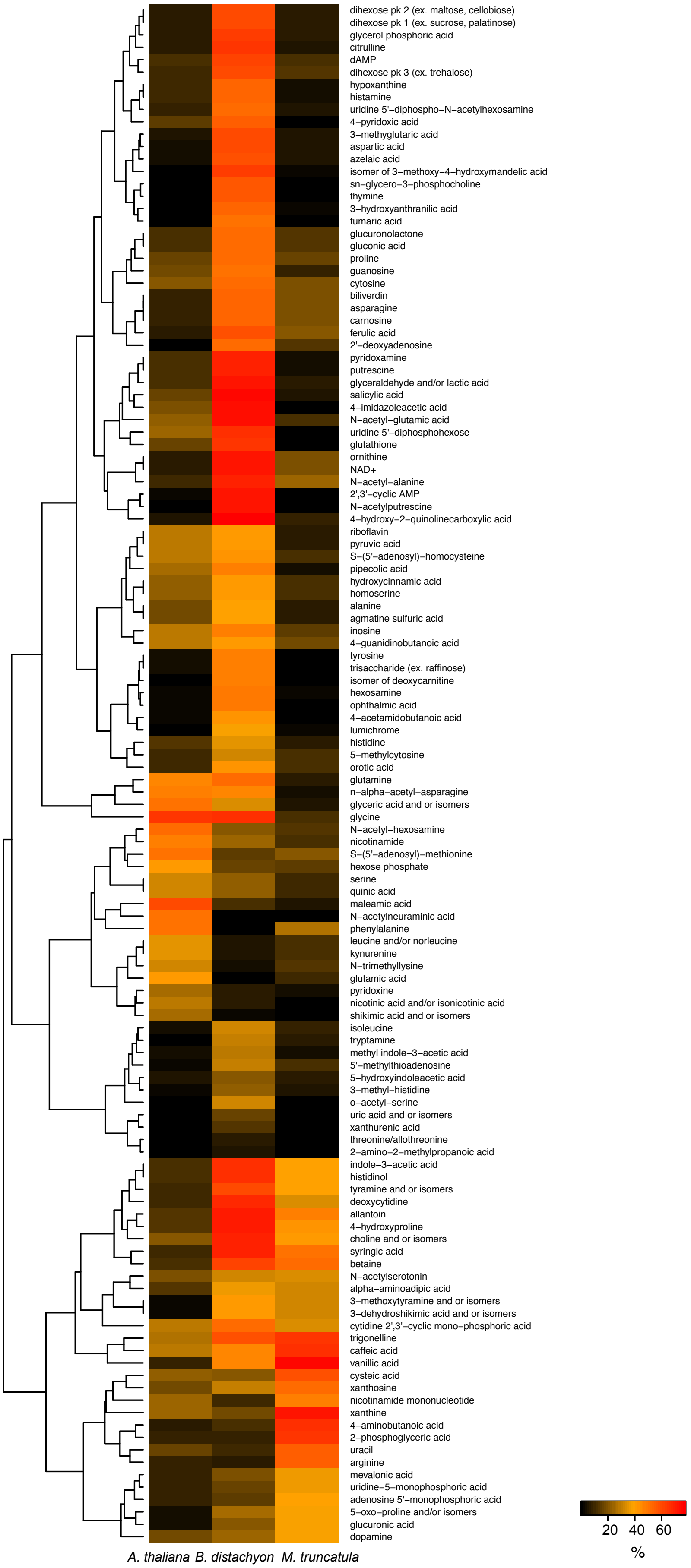

Supplementary Figure 6: Species-specific metabolites in tissues

Heatmap of shoot metabolites. Each cell represents an averaged value of normalized peak height of metabolites significantly different between plant species (Anova, $p < 0.05$ and post-hoc Duncan's multiple range test). Number of jars with 3-5 plants: 3-8 for each timepoint. One representative experiment out of three total is displayed. Heatmap of root metabolites is found in Figure 4, and PCA plots of tissues in Supplementary Figure 5.

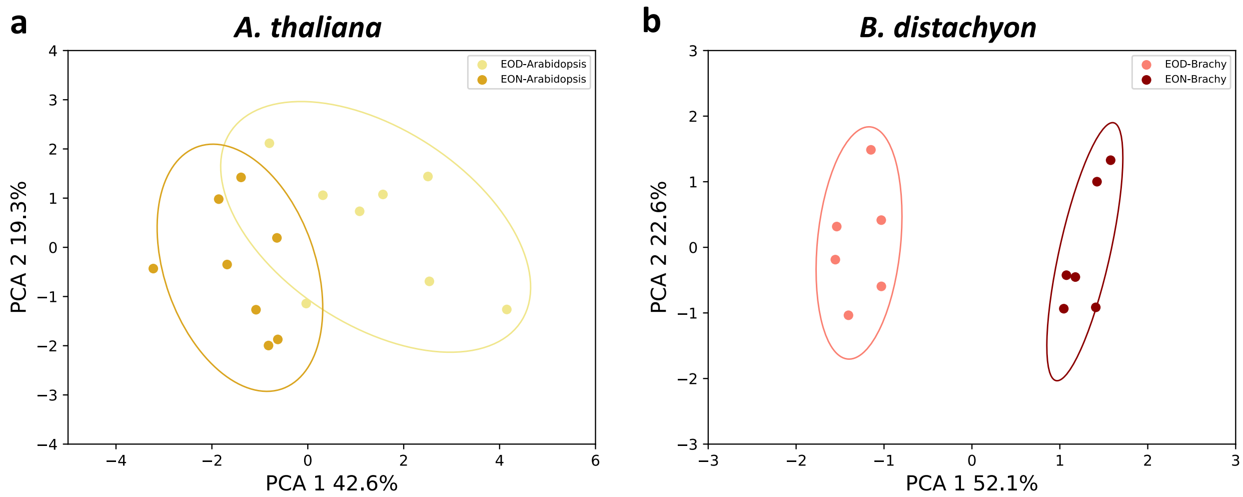

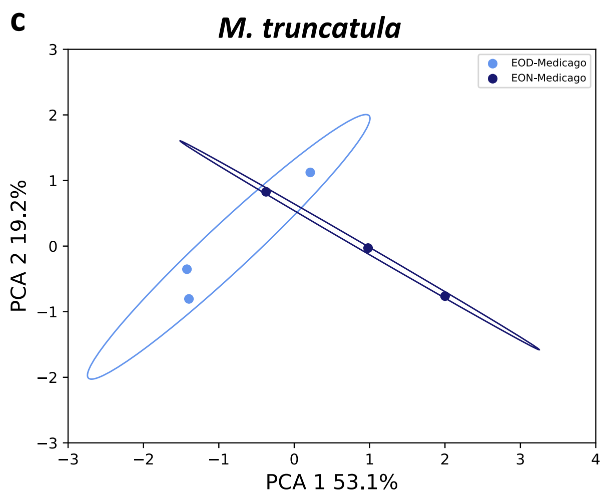

d

	changing	stable
Amino acids	8	**28**
Carbohydrates	3	4
Lipids	4	0
Nucleosides	14	8
Organic acids	14	7
others	7	4

Supplementary Figure 7: Species-specific metabolites in exudates

Principal component analyses of end of day and end of night exudate profiles of *A. thaliana* (**A**), *B. distachyon* (**B**), *M. truncatula* (**C**). **D**, Percentage of metabolites belonging to a specific metabolite class that are changing with a diurnal rhythm, or that remain stable. Blue, bold numbers indicate a number higher than expected compared to the overall percentage of metabolites in the dataset (Fisher's exact test).
