# Supplementary material for: The core metabolome and root exudation dynamics of three phylogenetically distinct plant species
Source: Nat Commun. 2023 Mar 24;14:1649. doi: 10.1038/s41467-023-37164-x (PMC10039077; doi:10.1038/s41467-023-37164-x)
Supplement: Supplementary file 3 — Description of Additional Supplementary Files [file 41467_2023_37164_MOESM3_ESM.pdf]

Description of Additional Supplementary Files:

Supplementary Data 1: Metabolomics data

Supplementary Data 2: LCMS parameters
