# Supplementary material for: The core metabolome and root exudation dynamics of three phylogenetically distinct plant species
Source: Nat Commun. 2023 Mar 24;14:1649. doi: 10.1038/s41467-023-37164-x (PMC10039077; doi:10.1038/s41467-023-37164-x)
Supplement: Supplementary file 6 — Reporting Summary [file 41467_2023_37164_MOESM6_ESM.pdf]

nature portfolio

Corresponding author(s): Sasse, Joelle; Northen Trent

Last updated by author(s): Feb 3, 2023

Reporting Summary

Nature Portfolio wishes to improve the reproducibility of the work that we publish. This form provides structure for consistency and transparency in reporting. For further information on Nature Portfolio policies, see our Editorial Policies and the Editorial Policy Checklist.

Statistics

For all statistical analyses, confirm that the following items are present in the figure legend, table legend, main text, or Methods section.

n/a	Confirmed	
☐	☒	The exact sample size (*n*) for each experimental group/condition, given as a discrete number and unit of measurement
☐	☒	A statement on whether measurements were taken from distinct samples or whether the same sample was measured repeatedly
☐	☒	The statistical test(s) used AND whether they are one- or two-sided<br>*Only common tests should be described solely by name; describe more complex techniques in the Methods section.*
☐	☒	A description of all covariates tested
☐	☒	A description of any assumptions or corrections, such as tests of normality and adjustment for multiple comparisons
☐	☒	A full description of the statistical parameters including central tendency (e.g. means) or other basic estimates (e.g. regression coefficient) AND variation (e.g. standard deviation) or associated estimates of uncertainty (e.g. confidence intervals)
☐	☒	For null hypothesis testing, the test statistic (e.g. *F*, *t*, *r*) with confidence intervals, effect sizes, degrees of freedom and *P* value noted<br>*Give P values as exact values whenever suitable.*
☒	☐	For Bayesian analysis, information on the choice of priors and Markov chain Monte Carlo settings
☒	☐	For hierarchical and complex designs, identification of the appropriate level for tests and full reporting of outcomes
☒	☐	Estimates of effect sizes (e.g. Cohen's *d*, Pearson's *r*), indicating how they were calculated

Our web collection on statistics for biologists contains articles on many of the points above.

Software and code

Policy information about availability of computer code

Data collection	LCMS instruments were run with MZmine.
Data analysis	Metabolite analysis was based on MZmine and Metabolite Atlas (https://github.com/biorack/metatlas). Stastistical tests were performed with Python.

For manuscripts utilizing custom algorithms or software that are central to the research but not yet described in published literature, software must be made available to editors and reviewers. We strongly encourage code deposition in a community repository (e.g. GitHub). See the Nature Portfolio guidelines for submitting code & software for further information.

Data

Policy information about availability of data

All manuscripts must include a data availability statement. This statement should provide the following information, where applicable:

- Accession codes, unique identifiers, or web links for publicly available datasets
- A description of any restrictions on data availability
- For clinical datasets or third party data, please ensure that the statement adheres to our policy

Provide your data availability statement here.

Human research participants

Policy information about studies involving human research participants and Sex and Gender in Research.

Reporting on sex and gender	NA
Population characteristics	NA
Recruitment	NA
Ethics oversight	NA

Note that full information on the approval of the study protocol must also be provided in the manuscript.

Field-specific reporting

Please select the one below that is the best fit for your research. If you are not sure, read the appropriate sections before making your selection.

☒ Life sciences ☐ Behavioural & social sciences ☐ Ecological, evolutionary & environmental sciences

For a reference copy of the document with all sections, see nature.com/documents/nr-reporting-summary-flat.pdf

Life sciences study design

All studies must disclose on these points even when the disclosure is negative.

Sample size	For jar experiments, three or more jars were prepared per treatment as biological replicates. In addition, the jars contained 3-5 plants (depending on the species) for additional biological replication. Each jar was sampled once per treatment and timepoint. Depending on the experiment, several timepoints per jar were sampled (e.g. end of day and end of night, or for the timecourse at different times after incubation). This sample size was chosen as previous experiments showed close grouping of biological replicates. For some experiments, a larger number of jars was prepared.<br>A set of three negative control jars (without plants, else same setup as biological replicates) was prepared to accurately determine the metabolite background in the experimental setup.
Data exclusions	Jars were excluded from analysis if they showed microbial contamination (by plating an aliquot of medium on LB plates), except for the jars that were set up nonsterile for the environmental experiment. Also, samples were excluded from analysis if they did not pass LCMS quality control (intensity of internal standard). Despite these criteria, the number of biological replicates never dropped below three.
Replication	For jar experiments, three or more jars were prepared per treatment as biological replicates. In addition, the jars contained 3-5 plants (depending on the species) for additional biological replication. Each jar was sampled once per treatment and timepoint. Depending on the experiment, several timepoints per jar were sampled (e.g. end of day and end of night, or for the timecourse at different times after incubation).
Randomization	LCMS sample injection order was randomized within one run. Roots, shoots, and exudates were run as separate groups, and LCMS quality control samples controlled for intensity and retention time shifts during the run.
Blinding	The jars of different treatments were given numbers for blinding. Some experimental setups did not allow for blinding (different plant species in jars with clearly distinct morphology, open/nonsterile vs closed/sterile jars).

Reporting for specific materials, systems and methods

We require information from authors about some types of materials, experimental systems and methods used in many studies. Here, indicate whether each material, system or method listed is relevant to your study. If you are not sure if a list item applies to your research, read the appropriate section before selecting a response.

Materials & experimental systems

n/a	Involved in the study
☒	☐ Antibodies
☒	☐ Eukaryotic cell lines
☒	☐ Palaeontology and archaeology
☒	☐ Animals and other organisms
☒	☐ Clinical data
☒	☐ Dual use research of concern

Methods

n/a	Involved in the study
☒	☐ ChIP-seq
☒	☐ Flow cytometry
☒	☐ MRI-based neuroimaging
